# A6 LYSOPHOSPHATIDYLCHOLINE (LPC) AND LYSOPHOSPHATIDIC ACID (LPA) INDUCE VISCERAL HYPERSENSITIVITY IN IRRITABLE BOWEL SYNDROME (IBS)

**DOI:** 10.1093/jcag/gwad061.006

**Published:** 2024-02-14

**Authors:** J Pujo, G De Palma, J Lu, G H Rueda, M Hall-Bruce, A Nardelli, F A Vicentini, E Verdu, S Vanner, S Collins, P Bercik

**Affiliations:** Farncombe Family Digestive Health Research Institute, McMaster University, Hamilton, ON, Canada; Farncombe Family Digestive Health Research Institute, McMaster University, Hamilton, ON, Canada; Farncombe Family Digestive Health Research Institute, McMaster University, Hamilton, ON, Canada; Farncombe Family Digestive Health Research Institute, McMaster University, Hamilton, ON, Canada; Farncombe Family Digestive Health Research Institute, McMaster University, Hamilton, ON, Canada; Farncombe Family Digestive Health Research Institute, McMaster University, Hamilton, ON, Canada; Farncombe Family Digestive Health Research Institute, McMaster University, Hamilton, ON, Canada; Farncombe Family Digestive Health Research Institute, McMaster University, Hamilton, ON, Canada; Gastrointestinal Diseases Research Unit, Queen’s University, Kingston, ON, Canada; Farncombe Family Digestive Health Research Institute, McMaster University, Hamilton, ON, Canada; Farncombe Family Digestive Health Research Institute, McMaster University, Hamilton, ON, Canada

## Abstract

**Background:**

Chronic abdominal pain is the key symptom in IBS. Gut microbiota produces a large variety of molecules that can regulate pain perception, including bioactive lipids. LPC and LPA are phospholipids, known to generate and maintain neurogenic pain in mammals, by directly activating multiple channels and G-protein coupled receptors on sensory neurons. LPC and LPA result from the conversion of phosphatidylcholine (PC), a major membrane-forming phospholipid in eucaryotes, as byproducts of inflammation. PC is also present in high concentration in eggs, meat and soybeans. It is unknown whether these two phospholipids play any role in chronic abdominal pain and whether they can be produced by the gut microbiota.

**Aims:**

To investigate whether 1) LPC and LPA are increased in stool of IBS patients, and 2) whether microbiota from IBS patients can produce LPC and LPA and lead to visceral hypersensitivity.

**Methods:**

Stool samples from 27 patients with IBS, collected both during periods of severe and minimal/no pain, were assessed for LPC and LPA levels by ELISA. Germ-free (GF) NIH Swiss and Swiss Webster mice (n=101) both sexes were colonized with fecal microbiota from IBS patients with either high or low fecal LPC and LPA levels, or microbiota from healthy controls. Mice were fed with control diet or diet enriched with PC diet (2g/kg of diet) for 5 weeks. Their visceral sensitivity was then assessed by measuring visceromotor responses to colorectal distension. The fecal samples were collected to measure the production of LPC and LPA

**Results:**

The fecal concentrations of LPC and LPA were higher in IBS patients when experiencing severe abdominal pain, compared to periods of minimal or no pain. Mice colonized with fecal microbiota from IBS patients with high fecal LPC and/or LPA displayed visceral hypersensitivity compared to mice colonized with microbiota from healthy controls, or microbiota from patients with low fecal LPC and/or LPA levels. However, this increase in visceral sensitivity was only found in mice fed with diet enriched with PC. Furthermore, the increased pain responses to colorectal distension correlated with higher concentrations of fecal LPC and LPA.

**Conclusions:**

Our data suggest that bacterial LPC and LPA is responsible for chronic abdominal pain in a subset of patients with IBS. Further studies are needed to identify the specific bacterial strains that produce these nociceptive phospholipids and precise mechanisms by which LPC and LPA induce visceral hypersensitivity.

**Funding Agencies:**

CIHRThe W.Garfield Weston Foundation

